# Biological response of an in vitro human 3D lung cell model exposed to brake wear debris varies based on brake pad formulation

**DOI:** 10.1007/s00204-018-2218-8

**Published:** 2018-05-10

**Authors:** Hana Barosova, Savvina Chortarea, Pavlina Peikertova, Martin J. D. Clift, Alke Petri-Fink, Jana Kukutschova, Barbara Rothen-Rutishauser

**Affiliations:** 1 BioNanomaterials Group, Adolphe Merkle Institute, University of Fribourg, Chemin des Verdiers 4, 1700 Fribourg, Switzerland; 2 Nanotechnology Centre, VŠB-Technical University of Ostrava, Ostrava, Czech Republic; 3 Laboratory for Materials-Biology Interactions, Empa, Swiss Federal Laboratories for Materials, Science and Technology, St. Gallen, Switzerland; 4 In Vitro Toxicology Group, Swansea University Medical School, Swansea, Wales UK; 5 Chemistry Department, University of Fribourg, Fribourg, Switzerland

**Keywords:** Brake wear particles, Toxicity, 3D model of the human alveolar epithelial tissue barrier, In vitro, Full-scale automotive brake dynamometer

## Abstract

Wear particles from automotive friction brake pads of various sizes, morphology, and chemical composition are significant contributors towards particulate matter. Knowledge concerning the potential adverse effects following inhalation exposure to brake wear debris is limited. Our aim was, therefore, to generate brake wear particles released from commercial low-metallic and non-asbestos organic automotive brake pads used in mid-size passenger cars by a full-scale brake dynamometer with an environmental chamber simulating urban driving and to deduce their potential hazard in vitro. The collected fractions were analysed using scanning electron microscopy via energy-dispersive X-ray spectroscopy (SEM–EDS) and Raman microspectroscopy. The biological impact of the samples was investigated using a human 3D multicellular model consisting of human epithelial cells (A549) and human primary immune cells (macrophages and dendritic cells) mimicking the human epithelial tissue barrier. The viability, morphology, oxidative stress, and (pro-)inflammatory response of the cells were assessed following 24 h exposure to ~ 12, ~ 24, and ~ 48 µg/cm^2^ of non-airborne samples and to ~ 3.7 µg/cm^2^ of different brake wear size fractions (2–4, 1–2, and 0.25–1 µm) applying a pseudo-air–liquid interface approach. Brake wear debris with low-metallic formula does not induce any adverse biological effects to the in vitro lung multicellular model. Brake wear particles from non-asbestos organic formulated pads, however, induced increased (pro-)inflammatory mediator release from the same in vitro system. The latter finding can be attributed to the different particle compositions, specifically the presence of anatase.

**Electronic supplementary material:**

The online version of this article (10.1007/s00204-018-2218-8) contains supplementary material, which is available to authorized users.

## Introduction

Traffic-related air pollutants can be released into the environment by exhaust and non-exhaust emissions (Grigoratos and Martini 2015). Exhaust emissions derive mainly from combustion processes by, e.g., diesel or gasoline cars, whereas non-exhaust particles can be generated from wear of brakes, tyres, clutches, and road surfaces (Thorpe and Harrison 2008). Due to stricter controls of exhaust emissions, the relative contribution of non-exhaust sources to traffic-related emissions is increasing. Recent studies have shown that brake wear particles can contribute up to 55% of the total non-exhaust traffic-related PM_10_ (particulate matter ≤ 10 µm) emissions and up to 21% of total traffic-related PM_10_ emissions in urban areas (Harrison et al. 2012; Lawrence et al. 2013).

Braking systems are one of the critical elements for traffic safety. The formulation of friction composites is important for the proper function of the complete system. Automotive friction composites are multicomponent formulas consisting of more than ten components; however, producers can use up to 2000 different components for specific composites (Filip et al. 1997). The formulation and amount of each component are considered proprietary by manufacturers and thus make the characterisation of commercially available brake pad materials challenging. However, regular automotive brake pads contain five basic types of components in general, i.e., fibers (e.g., various metals, carbon, glass, and Kevlar) which provide mechanical strength, abrasives (e.g., aluminium and iron oxides, quartz (silicon oxide), and zirconium oxide) to increase friction, lubricants (e.g., graphite and various metal sulphides) to stabilize the friction properties at high temperature, fillers (e.g., barite, calcite, and mica) to improve manufacturability, and binders (e.g., phenolic resin) to maintain the structural integrity under mechanical and thermal stress (Thorpe and Harrison 2008). Kukutschova et al. (2011) studied airborne wear particles released from low-metallic commercial friction composites and detected the emission of nano-sized particles with a diameter around 20 nm at concentrations about several millions per cubic centimeter. Emitted airborne wear particles were separated into several nano- and micro-sized fractions and 20 nm particles were detected to be attached to the surface of larger particles. Furthermore, high temperatures and pressures during braking can evoke the transformation of some raw materials [Antimony trisulfide (Sb_2_S_3_)] into new compounds [Antimony pentoxide (Sb_2_O_5_), Laves Sb_2_Fe phase], which may also pose a potential risk to human health (Uexküll et al. 2005). Garg et al. (2000) estimated the total brake wear for a small car in range of 3.2–8.8 mg/km according to the type of tested pad among wear debris generated from “non-asbestos formulations”. Particle sizes range from 0.5 to 10 µm, and the average size is around 6 µm. Furthermore, 35% of brake pad mass loss was emitted as airborne particles with 86% of the airborne particles smaller than 10 µm (PM_10_) and 63% smaller than 2.5 µm in diameter (PM_2.5_) (Sanders et al. 2003). Hagino et al. (2016) observed brake wear particle emission from non-asbestos organic brake pads in a range of 0.04–1.4 mg/km/vehicle for PM_10_ and 0.04–1.2 mg/km/vehicle for PM_2.5_. Brake wear particles were produced by a brake wear dynamometer with a constant volume sampling system using both passenger cars and middle-class trucks. The proportion of brake wear particles emitted as airborne was 2–21% of the total mass of overall wear debris (Hagino et al. 2016).

Traffic intensity is one of the most important determinants of ambient, anthropogenic PM concentration, and people living in cities and near major traffic routes are particularly affected by high levels of PM pollution (Wiedensohler et al. 2000). Ambient PM was found to cause adverse health effects associated with increased pulmonary and cardiovascular mortality (Peters et al. 1997; Schulz et al. 2005; Sun et al. 2010). Studies have demonstrated that particle size affects particle deposition in the respiratory tract (Kumar et al. 2013; Pope et al. 2002; Samet et al. 2000). Coarse particles in the size range of several µm are mainly deposited in the upper respiratory tract, while ultrafine particles (< 100 nm) can penetrate into the deep lung (Stone et al. 2017). Particles that are inhaled and deposited on the alveolar lung cell surface can induce oxidative stress and (pro-)inflammatory reactions leading to more severe lung diseases such as chronic obstructive pulmonary disease (Balakrishna et al. 2009; Karlsson et al. 2005; Oberdörster et al. 2005). In vivo studies have shown that ultrafine particles can translocate across the air–blood barrier into the bloodstream and thus reach secondary organs including the liver, kidneys, and brain (Geiser and Kreyling 2010; Oberdörster et al. 2005; Tjalve and Henriksson 1999). The World Health Organisation reported that adverse health effects (i.e., respiratory, cardiovascular morbidity, and lung cancer) of inhalable PM can occur due to exposure over both short (hours, days) and long (months, years) time frames (World Health Organisation 2013).

A considerable fraction of brake wear particles have diameters < 100 nm (Garg et al. 2000; Mathissen et al. 2011), thus concerns regarding their potential adverse health effects associated with their inhalation have been raised. Only few studies have focused on potential health risks of brake wear particles. Gasser et al. (2009) exposed lung cells (A549 alveolar epithelial monoculture) directly to freshly emitted wear particles. Their results suggest that brake wear particles derived from a “full stop” braking pattern caused a small increase in the (pro-)inflammatory response (interleukin-8 release) in the lung epithelial cells after 24 h post-exposure. This finding was most likely associated with increased concentrations of organic compounds and carbon black.

The aim of the current study was to assess a possible correlation of brake pads composition with the physico-chemical characteristics of the released particles and the impact of these particles upon a human 3D lung model in vitro mimicking the human alveolar epithelial tissue barrier (Rothen-Rutishauser et al. 2005). First, investigation of the physico-chemical properties of brake wear debris particles, dependent on the initial brake pad formulation, was performed. Second, exposure of the particles using a pseudo-air liquid interface (pseudo-ALI) approach, as previously described (Endes et al. 2014), with the lung model consisting of A549 (alveolar epithelial type II cells) with human primary macrophages (MDMs) and dendritic cells (MDDCs) was applied to investigate the potential health risks corresponding to the brake pad formulation as well as size fraction of brake wear particles at realistic lifetime doses.

## Materials and methods

### Sample preparation

Brake wear particles were generated and collected according to a procedure simulating urban driving conditions (speed below 80 km/h, rotor temperature between 200 and 500 °C). Brake dynamometer tests were performed using the full-scale brake dynamometer model M2800 (Link Engineering Co., USA, shown in Supplementary Fig. 1a) with the brake system closed in an environmental chamber. The chamber contains the entire front wheel with a disc and caliper and it also contains a wind tunnel simulating a corresponding airflow. The chamber was cleaned before each test and the non-airborne wear debris were collected by sweeping the surfaces of a plastic bin placed at the bottom of the chamber. Airborne particles were captured and separated by size-resolved sampling into different size fractions using the aerosol sampling system NANO-ID SELECT (PMS Inc., USA) including 12 channels with the size range 0.001–35 µm. The investigated size fractions (2–4, 1–2, and 0.25–1 µm) were collected on the glass side. The controlled filtered air had a flow capacity of 25 L/min during the testing procedure. The brake pads used for the generation of wear particles were sorted based on the initial brake pad formulation. Brake pads with a ‘low-metallic’ formulation (i.e., below 3 wt% of metallic components) were used for the generation of non-airborne fraction samples [marked as ‘non-airborne low-metallic’ sample (nLM)] and for the generation of different size fractions (2–4, 1–2, and 0.25–1 µm) of brake wear particles [marked as ‘size fractions of low-metallic’ sample (sfLM)]. Brake pads with non-asbestos organic (NAO) formulation were used for generation of the non-airborne fraction, size fractions of NAO could not be collected and size-sorted because of high carbonaceous content revealing the equipment limitations.

### Sample characterisation

All samples were analysed using SEM (MIRA 3, TESCAN, Czech Republic) with EDS. The powder samples were applied onto the carbon tape and spin-coated with 4 nm layer of gold to obtain effective electrical conductivity. The secondary electron (SE) mode was used for observing all samples by either SE or InBeam detector. EDS detector was used to determine the elemental composition.

Phase analysis of all powder samples was performed by Raman microspectroscopy using Smart Raman microscopy (System XploRA™, HORIBA Jobin Yvon, France). Raman spectra were acquired with a 532 nm excitation laser source, and 1200 grooves/mm grating.

The brake wear particles’ morphology was investigated via transmission Electron microscopy (TEM, Fei Technai Spirit, USA) operating at 120 kV and equipped with a Veleta CCD camera (Olympus, Japan). Particles were suspended in deionized water, sonicated for 90 min and a drop of 20 µL was pipetted on TEM copper grids.

### Endotoxin content

Endotoxin content of each non-airborne sample (i.e., nLM, NAO) was assessed. Samples at concentrations of 2, 1, and 0.5 mg/mL suspended in endotoxin-free water were analysed. Quantification of the endotoxin content was performed after 1 h of incubation (at 37 °C, 5% CO_2_) using the Pierce™ LAL Chromogenic Endotoxin Quantitation Kit (cat. no.: 88282; Thermo Scientific, USA). No endotoxin content was detected (value < 0.5 EU/mL) in all samples.

### 3D human alveolar lung model

The co-culture model consisted of human epithelial type-II cells (A549 cell line), combined with primary human blood monocyte derived macrophages (MDMs) and dendritic cells (MDDCs) as described by Lehmann et al. (2010). Detailed description can be found in Electronic Supplementary material (SI).

Briefly, cells were cultured in Roswell Park Memorial Institute medium 1640 supplemented with 10% fetal bovine serum, 1% penicillin/streptomycin, and 1% l-glutamine (sRPMI, all Gibco, USA) at 37 °C, 5% CO_2_. Epithelial cells were seeded at a density of 2.4 × 10^5^ cells/cm^2^, in BD Falcon cell culture inserts. Inserts were placed in culture plates (BD Biosciences, Switzerland) and cells were grown for 5 days under submerged conditions. Peripheral human blood monocytes were isolated from human blood buffy coats (Blood Donation Service, Bern University hospital, Bern, Switzerland), as previously described by Lehmann et al. (2010) using CD14^+^ MicroBeads (Miltenyi Biotec GmbH, Germany) according to the manufacturer’s protocol. Monocytes were cultured for 7 days prior to assembling the co-culture. For the cell differentiation the growth factors [GM-CSF and IL-4 (both [10 ng/mL]) for MDDCs and M-CSF [10 ng/mL] for MDMs] were applied to the medium. The co-culture models were assembled, as previously described (Blank et al. 2007). After 24 h under submerged conditions the co-cultures were transferred to the ALI conditions, by removing the medium in the upper chamber and replacing the medium in the lower chamber with 1.2 mL of fresh culture medium in 6-well plate or 0.6 mL in 12-well plate, respectively. The cells were then exposed to air for an additional 24 h prior the exposures being performed.

### Lung cell exposures

The particles were directly suspended in sRPMI medium and sonicated for 90 min prior exposure. Co-cultures grown in 6-well plate inserts at the ALI were then exposed to 100 µL suspended non-airborne brake wear particles on the apical side (referred as pseudo-ALI approach as described by Endes et al. 2014). The concentrations of non-airborne brake wear particles (i.e., samples nLM and NAO) were 2, 1 and 0.5 mg/mL, considering that majority of the particles sediments on the cells within 24 h and taking into account the effective growth area of the insert (4.2 cm^2^), the particle deposition corresponds to ~ 48, 24 and 12 µg/cm^2^, respectively. Cells in 12-well inserts were exposed to 50 µL of suspension of sfLM because of the limited particle mass received during the 16 h braking cycles, the concentration for all sfLM used was 33 µg/mL, which correspond with deposition ~ 3.7 µg/cm^2^ (effective growth area of 12-well inserts is 0.9 cm^2^).

### Cell morphology

After the 24 h incubation period, the co-cultures were fixed for 15 min in 3% paraformaldehyde (PFA) in phosphate buffered saline (PBS), treated with 0.1 M glycine in PBS for 15 min, and subsequently treated with 0.2% Triton X-100 in PBS for 15 min to permeabilise the cell membrane. Phalloidin rhodamine (R-415; Molecular Probes, Life Technologies, Switzerland) was used to stain the F-actin cytoskeleton (1:50 dilution), while 4′,6-diamidin-2-fenylindol (DAPI, [100 mg/mL], Sigma-Aldrich, Switzerland) stained nucleus at a 1:50 dilution. Finally samples were embedded in Glycergel (DAKO Schweiz AG, Switzerland). An inverted laser scanning confocal microscope (LSM 710, Zeiss, Germany) was used for the sample visualisation. Image processing was performed using the restoration software IMARIS (Bitplane AG, Switzerland).

### Cell viability (propidium iodide assay)

To investigate cell viability, cells were detached from the insert with trypsin–EDTA (0.05%, Gibco, USA) using the method previously described by Clift et al. (2017). Exposed cells were incubated with propidium iodide (PI) (Annexin-V-FLUOS staining kit, Roche Diagnostics, Switzerland) to stain necrotic cells for 15 min at RT and then immediately analysed by flow cytometry [LSR Fortessa (3 laser, 4-blue-2-red-2-violet (405 nm (violet); 488 nm (blue); 640 nm (red)) BD Biosciences, Basel, Switzerland)]. Untreated cells were incubated at − 80 °C for 1 h as a positive control to induce necrosis. The detailed gating strategy is explained in the SI.

### Biochemical analysis

- Oxidative stress response
  The concentration of total glutathione (GSH) was determined with a diagnostic glutathione assay kit (Cayman Chemical Company, USA), following the manufacturer’s instructions. GSH values are reported relative to the total amount of protein of each sample determined by the Pierce bicinchoninic acid (BCA) protein assay kit (Pierce Protein research Products, Thermo Scientific, USA). Cells exposed to 135 mM tert-butyl hydrogen peroxide (tBHP) served as the positive control.
- Cytokine/chemokine quantification
  The (pro-)inflammatory response was measured by quantifying the amount of the (pro-)inflammatory mediators released into the lower medium chamber, e.g., tumor necrosis factor α (TNF-α), interleukin-1β (IL-1β) and interleukin-8 (IL-8) via enzyme-linked immunosorbent assay (ELISA) using the commercially available DuoSet ELISA Development Kit (R&D Systems, Switzerland) according to the supplier’s protocol. Co-cultures treated basolaterally with Lipopolysaccharide (LPS from *Escherichia coli* at [1 µg/mL]) for 24 h acted as the positive control. No particle interference was observed at any of the applied concentration, each ELISA kit was tested separately (data not shown).

### Statistical analysis

All data is presented as the mean ± standard error of the mean (STEM). A total of three independent experiments (*n* = 3) have been performed for all endpoints. Statistical analysis was performed using GraphPad Prism 6 (GraphPad Software Inc., USA). Assuming normal distribution of the data sets, a parametric one-way analysis of variance (ANOVA) followed by Dunnett’s multiple comparison test was performed. Results were considered significant if *p* < 0.05.

## Results

### Sample characterisation

**Fig. 1:**
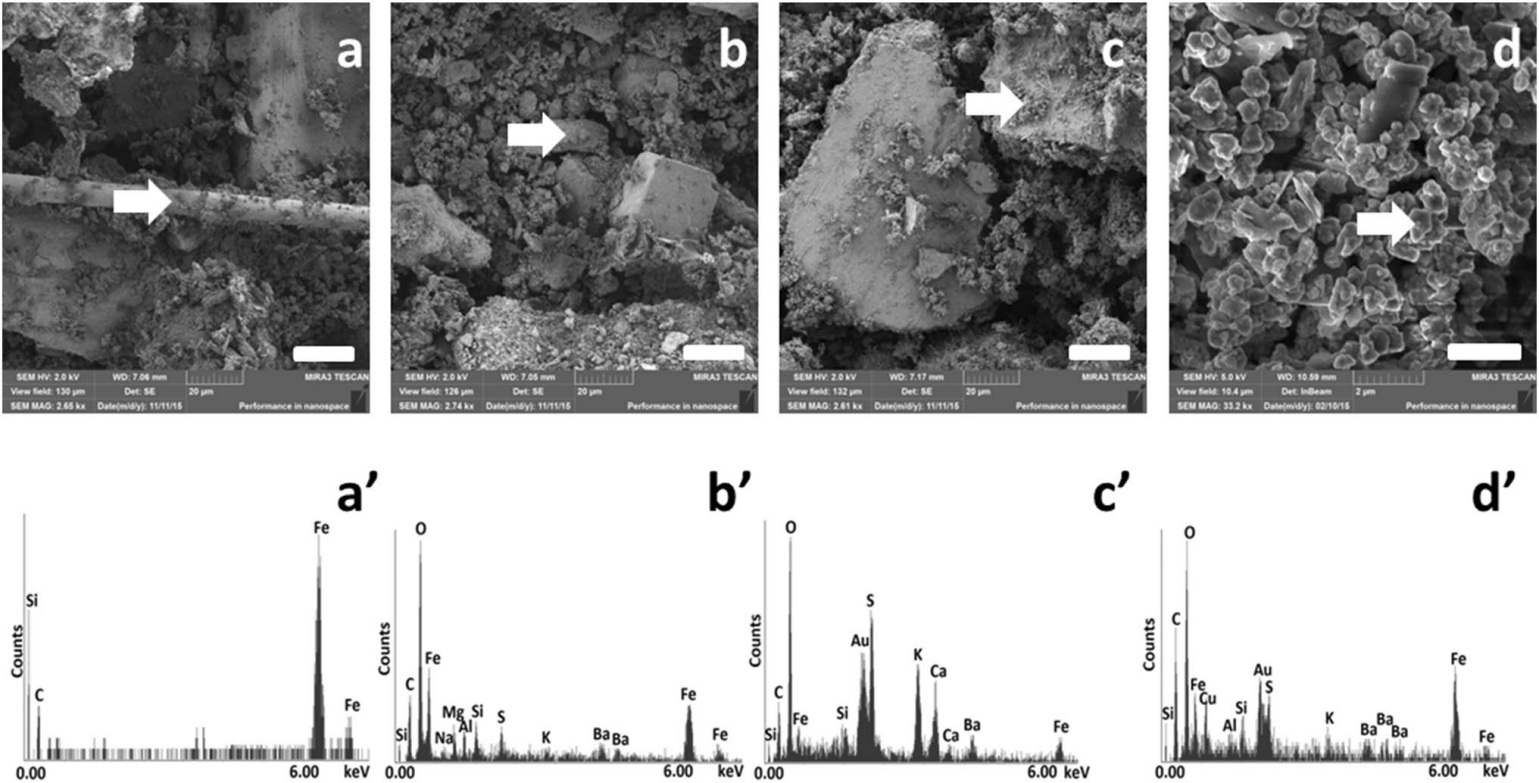
SEM micrographs of nLM (**a, b**) with corresponding EDS spectra (**a**’, **b**’), NAO sample (**c**) with corresponding EDS spectrum (**c**’) and 1–2 µm size fraction of sfLM sample (**d**) with corresponding EDS spectrum (**d**’). White arrow refers to the spot of EDS scanning

SEM images of both non-airborne samples (nLM and NAO) and 1–2 µm size fraction of low-metallic sample are shown at Fig. 1a–d, respectively. SEM micrographs show the heterogeneity of the samples. Rod-like particles with smaller agglomerates attached on the surface were observed (Fig. 1a). EDS elemental analysis proved the iron origin of the rod in the nLM sample (Fig. 1a’). No visual difference between the two non-airborne samples (nLM and NAO) was observed. Agglomerates of smaller particles in the sub-micron size range on the surfaces of the bulk materials were visualised in both samples (Fig. 1b, c). The importance of particle separation by size was indicated (Fig. 1d); a homogenous sample has been observed in the 1–2 µm fraction of sfLM sample. All elements detected by EDS are summarized in Table 1; however, carbon, oxygen, sulphur, potassium, silicon and iron were detected in all samples. The presence of gold present in EDS spectra originates from spin-coating of the sample.

**Table 1:** Summary of all applied samples together with their biological response (black arrows with star (↑*) show significant increase, simple arrows (↑) show tendency)

Brake formulation		Low-metallic				Non-asbestos organic
Particle fraction		Non-airborne	2–4 µm	1–2 µm	0.25–1 µm	Non-airborne
Elemental analysis (EDS)		Al, Ba, C, Fe, K, Na, O, S, Si	Al, Ba, C, Ca, Cu, Fe, K, Mg, O, S, Si, Ti			Ba, Ca, C, Fe, K, O, S, Si
Phase analysis (Raman microspectroscopy)		Amorphous carbon, graphite, Fe_2_O_3_, Fe_3_O_4_	Amorphous carbon, graphite, SiC			Amorphous carbon, graphite, TiO_2_, Fe_2_O_3_
Exposure scenario		Mild occupational exposure	Ambient conditions			Mild occupational exposure
Cytotoxicity		↑	↑	↑	↑	↑
Oxidative stress		–	–	–	–	–
(Pro-)inflammatory mediators	IL-8	–	–	–	–	↑*
	IL-1β	↑	–	–	–	–
	TNF-α	–	–	–	–	–

Elemental analysis was performed using energy-dispersed spectroscopy (EDS); cell viability was assessed by measuring propidium iodide (PI) positive cells; oxidative stress was investigated by measuring total glutathione (GSH) presented as relative to total protein; (pro-)inflammatory response was assessed by measuring tumor necrosis factor-α (TNF-α) and interleukin-1β and -8 (IL-1β and IL-8)

**Fig. 2:**
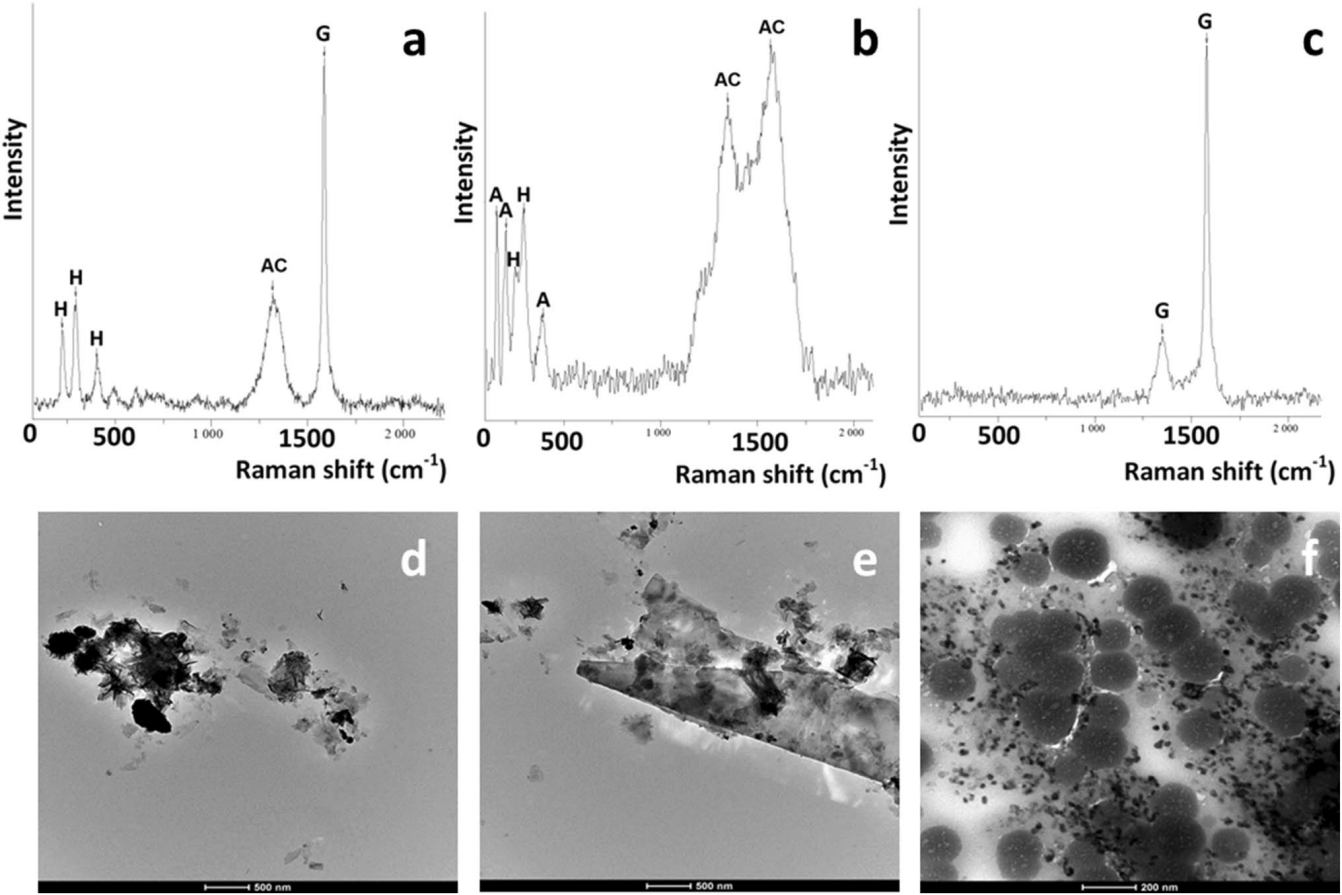
Representative Raman spectra of nLM sample (**a**), NAO sample (**b**) and sfLM sample (**c**) with characteristic bands for hematite (H), amorphous carbon (AC), graphite (G), and anatase (A) and TEM micrographs of nLM (**d, e**) and 2–4 µm sfLM sample (**f**)

Raman microspectroscopy confirmed the presence of carbon (either amorphous carbon or graphite) in all samples. Furthermore, the presence of hematite (α-Fe_2_O_3_), graphite and amorphous carbon in the nLM sample (Fig. 2a), silicon carbide (SiC) (spectrum not shown), anatase (TiO_2_), and hematite (α-Fe_2_O_3_) in NAO sample (Fig. 2b) and graphite in sfLM sample (Fig. 2c) was revealed.

The Raman spectra of the nLM (Fig. 2a) and NAO (Fig. 2b) samples show the presence of α-Fe_2_O_3_ with significant bands at 225, 296 and 405 cm^−1^. Narrow band at ~ 1580 cm^−1^ in nLM and sLM samples confirms the presence of graphite (Wang et al. 1990), while broad bands at ~ 1355, and ~ 1530 cm^−1^ demonstrate the amorphous carbon content in all samples (Scheibe et al. 1995). Bands at ~ 152, ~ 201, and ~ 391 cm^−1^ proof the presence of TiO_2_ in form of anatase (Choi et al. 2005) in NAO sample (Fig. 2b).

TEM micrographs (Fig. 2d–f) confirm the heterogeneity of the samples. Different particle shapes and sizes can be observed, such as needle-like particle structures at sub-micron sizes (Fig. 2d). Smaller particles can agglomerate and adhere to the surface of bulk materials (Fig. 2e). The presence of small particles (nm size range) besides bigger (> 200 nm diameter) carbon-based spheres can be also observed (Fig. 2f).

### Particle exposure to cell surface

The particles suspended in sRPMI were applied on the surface (pseudo-ALI approach) of the cells cultured at ALI at different concentrations of non-airborne brake wear particles (i.e., 2, 1 and 0.5 mg/mL), resulting in a very thin layer (~ 230 µm) of the suspension with particle deposition of around ~ 48, 24 and 12 µg/cm^2^, respectively. The concentration for all sfLM (33 µg/mL) corresponds with a deposition of ~ 3,7 µg/cm^2^. It was not possible to increase this concentration because of limited particle mass received during the sampling process.

### Cell viability and morphology

**Fig. 3:**
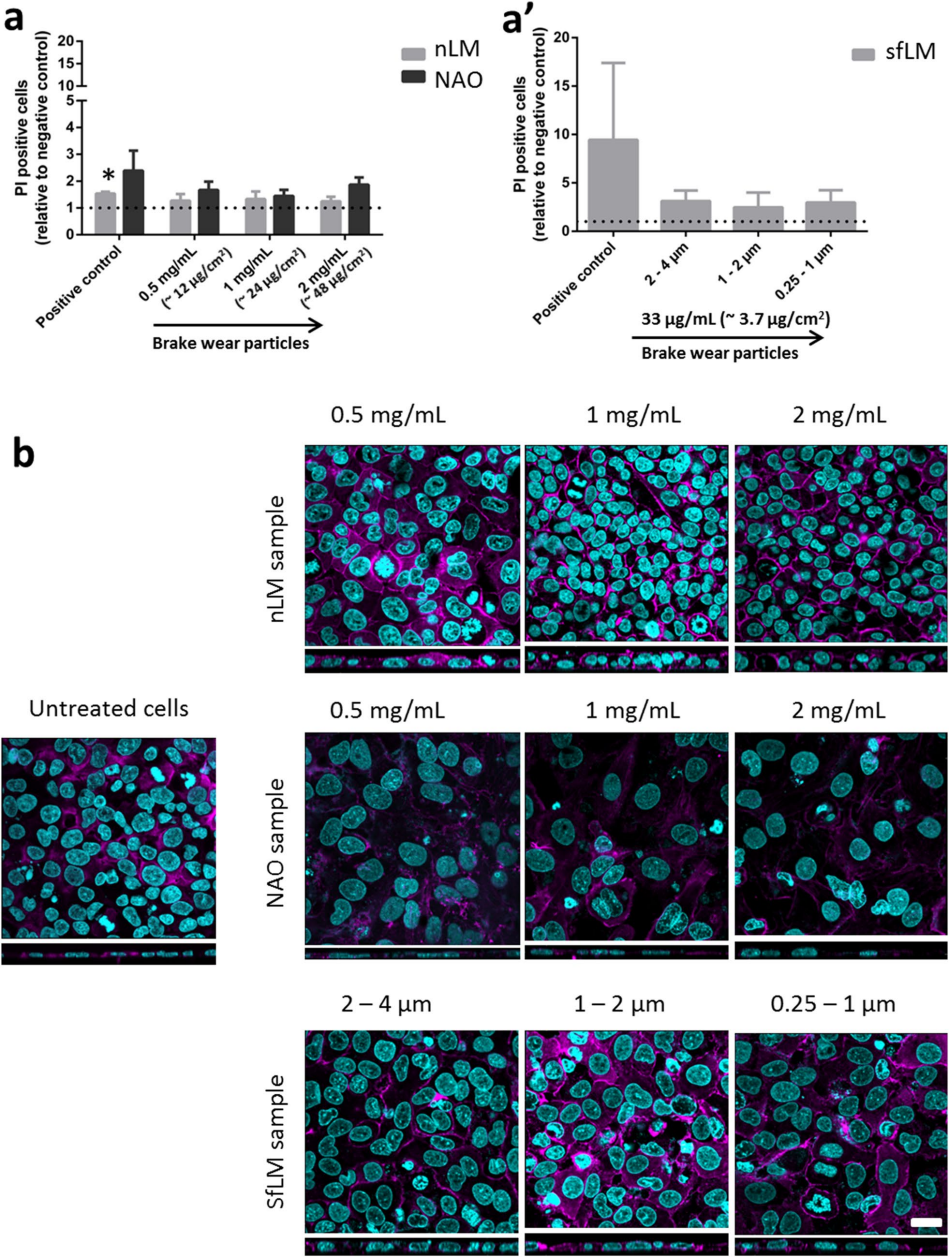
Cell viability (**a**) and cellular morphology (**b**) assessment in lung cell cultures exposed to brake wear particles. Cell viability (PI staining) was determined by comparison of brake wear particle exposed cell samples to the negative control (untreated cells) for nLM and NAO samples (**a**) and for sfLM samples (**a**’) (results shown fold increase over untreated cells, dashed line *y* = 1, i.e., indicating the level of negative control). Positive control: Untreated cells incubated at − 80 °C for 1 h to induce necrosis. Data are presented as mean ± standard error of the mean (*n* = 3). Data marked as (*) were considered statistically significantly increased compared to negative control (*p* < 0.05). Confocal laser scanning microscopy images of co-cultures (**b**) exposed to nLM, NAO, and sfLM samples. Purple color shows the F-actin cytoskeleton, blue color shows nuclei. Scale bar is 20 µm

Flow cytometry was used to detect the necrotic cell population. The fold increase of cells positively stained by PI compared to untreated cells is shown in Fig. 3a. No significant change in viability was observed across all samples analysed. However, an increase for PI positive cells for the NAO exposed samples was seen. Cell morphology was investigated by laser scanning microscopy (LSM) and no difference compared to untreated cells was observed in all ‘low-metallic’ samples (i.e., nLM and sfLM). However, morphology change was seen for the cytoskeleton of cells exposed to NAO (see Fig. 3b). The decrease in cell layer thickness could be a result of the decreased number of cells observed. Furthermore, most cells became disorganized, while losing cellular contacts and regular shape.

### Oxidative stress and (pro-)inflammatory response

**Fig. 4:**
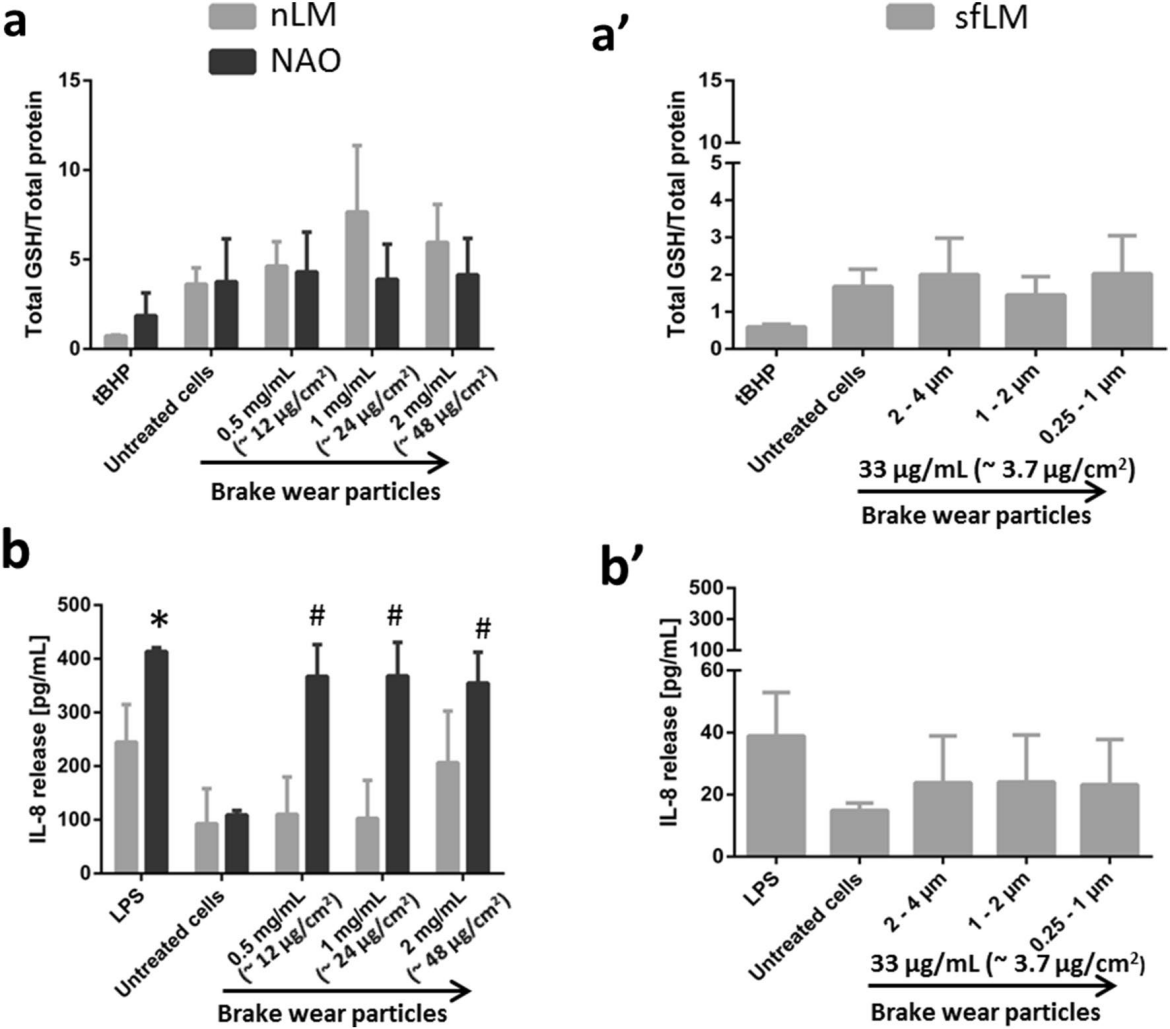
Oxidative stress and (pro-)inflammatory response in lung cells exposed to brake wear particles. Oxidative stress levels in lung cell cultures following exposure to nLM and NAO samples (**a**) and to sfLM sample (**a**’). Quantification of IL-8 release for nLM and NAO samples (**b**) and for sfLM sample (**b**’). Data are presented as mean ± standard error of the mean (*n* = 3). Data marked as (*) were considered statistically significantly increased compared to negative control (*p* < 0.05). *LPS* lipopolysaccharide, *tBHP tert*-Butyl hydroperoxide

The total amount of glutathione (GSH) relative to total protein was analysed to evaluate oxidative stress (Fig. 4a). No significant changes (*p* > 0.05) to the oxidative stress status in all cell cultures were seen. A decrease in total glutathione levels was only observed for the positive control (*t*BHP).

The cytokine release of IL-8 (Fig. 4b), TNF-α (Supplementary Fig. 1b) and IL-1β (Supplementary Fig. 1c) was determined to evaluate the (pro-)inflammatory response of the co-culture model. A statistically significant increase (*p* < 0.05) was observed in regards to the release of IL-8 following NAO exposure at all concentrations and the positive control.

## Discussion

The increase of brake wear particulate emissions released into the environment correlates with increasing traffic density. The aim of this study was to correlate the physico-chemical characteristics of particles released from different brake pads with their possible adverse effects in vitro using a 3D human alveolar model. The brake pads for small passenger cars used for the generation of wear particles are currently commercially available on the US and European market.

Hereby we presented a combination of methods which are necessary to obtain overall information about such a heterogeneous material leading to possible prediction of adverse health effects. All the results obtained in this study are summarized in Table 1.

### Brake wear debris

Particles settled on the bottom of the environmental chamber of the dynamometer are presented as ‘non-airborne’, while ‘airborne’ particles were sorted into different size fractions using an aerosol sampling system within the environmental chamber of the dynamometer. However, a previous study (Kukutschová et al. 2011) revealed micro-sized particles present in the form of clusters in the non-airborne fraction and nano-sized particles attached on the surface of larger micro-sized airborne particles in all size fractions as separated using an impactor. Brake pads with ‘low-metallic’ formulation (i.e., nLM and sfLM samples) typically consist of organic compounds (mainly phenolic resin) mixed with a small amount of metallic components (up to 3% by mass), providing high friction and good braking capacity at high temperatures. On the other hand, brake pads with ‘non-asbestos’ formulation (i.e., NAO sample) are relatively soft, contain thermally less stable components and, therefore, lose the braking capacity at high temperatures, and thus release more wear debris than other types of brake pads (Grigoratos and Martini 2015; Lemen 2004).

As previously shown, heat and high pressure generated by friction processes result in morphological and chemical composition differences of released wear particles compared to the original brake formulation (Blau and Meyer Iii 2003; Filip et al. 1997; Kukutschová et al. 2009; Österle et al. 2001). EDS results corresponding with Raman microspectroscopy results showed the presence of carbonaceous materials such as amorphous carbon and graphite in all samples. Amorphous carbon is likely produced by oxidative wear of phenolic resin, condensation and subsequent deposition from the volatiles, on the other hand graphitic structures in the form of fine or coarse particles are most probably deposited from abrasive wear (Kukutschová et al. 2011). Since the exact formulation of each brake pad is manufacturer’s proprietary, the comparison of the original brake pad with obtained brake wear particles was not possible. However, iron or iron oxide powder, together with quartz, zirconium, and aluminium oxide are the most common abrasive constituents (Grigoratos and Martini 2015; Kazimirova et al. 2016). Silicon carbide detected in sfLM sample is used as an abrasive material for brake pads improving the mechanical properties at high temperatures (Krenkel et al. 2002). In addition, the first composites applied in brake pad formulations were copper-coated silicon carbide composites (Kennedy et al. 1997), which could explain the presence of copper in EDS spectrum in the sfLM sample. Another material detected by Raman microspectroscopy was anatase (TiO_2_) in NAO sample. The TiO_2_ probably originates from the original brake pad formula, which corresponds with earlier studies in terms of the trace elements observed in the brake wear dust (Garg et al. 2000; Hildemann et al. 1991; Sanders et al. 2003; Uexküll et al. 2005; Westerlund and Johansson 2002) as well as other elements like aluminium, barium, calcium, iron, magnesium, potassium, and sodium detected by EDS. It is important to mention that the detection limit of Raman microspectroscopy is in the range of the size of the laser spot, i.e., particles < 500 nm are most likely undetectable. Nevertheless, Raman microspectroscopy is a point analysis and has very good potential in the study of such heterogeneous samples. Additionally, EDS analysis showed a similar composition of all the tested samples with minor differences proving the deviations in the initial formulation of tested brake pads. Raman microspectroscopy also revealed the same basic composition of the brake wear debris, which is in good accordance with EDS analysis. Amorphous carbon was found in all studied samples by Raman microspectroscopy, which mostly originates from phenolic resin burnish and creates thin film of amorphous carbon which covered almost entire sample (Kukutschová et al. 2011; Peikertová et al. 2013).

SEM and TEM micrographs showed the heterogeneity of the samples, as well as the strong adherence of smaller agglomerates to the larger particles as already shown (Kukutschová et al. 2011). The adherence of the nano- and sub-micron particle agglomerates to larger particles can be also observed by TEM. Reason for this finding is hypothesized, that the smaller particles re-agglomerate to the larger particles during the drying process of TEM samples.

Particle size affects their deposition in the respiratory tract (Kumar et al. 2013; Pope et al. 2002; Samet et al. 2000). Inhaled fine and ultrafine particles may penetrate deep into the lungs causing oxidative stress and a (pro-)inflammatory effect; PM_2.5_ has been reported to penetrate into the small airways as well as the lung parenchyma, and can eventually translocate into the bloodstream (Geiser and Kreyling 2010; Oberdörster et al. 2005). Hagino et al. (2016) estimated that the realistic average mass concentration of ultrafine particles (mobility diameter < 100 nm) in an urban environment is up to 10 µg/m^3^. An upper limit for workplace nanoparticle mass concentrations of 5 mg/m^3^ has been recommended by the Occupational Safety and Health Administration (OSHA, USA) standard for respirable nuisance dust (averaged over an 8 h work shift), which corresponds to daily alveolar mass dose of 0.13 mg/cm^2^ (clearance is neglected; 100% biopersistence is assumed). Hence, the maximum lifetime dose accumulated by a workers’ occupational life is up to 420 mg/cm^2^ (i.e., assumed to be 5 workdays per week for 50 weeks per year over 45 years; with 70% clearance).

Brake wear particles were roughly estimated to contribute to ambient PM_10_ concentrations up to 4 µg/m^3^ (Amato et al. 2009; Harrison et al. 2012; van der Gon et al. 2007; Wåhlin et al. 2006), so according to the assumption above, lifetime exposure of brake wear particles correspond to ~ 2.4 µg/cm^2^ under realistic ambient conditions, while in ‘worst case scenario’ can reach up to ~ 180 µg/cm^2^. The concentration applied for NAO and nLM samples (12–48 µg/cm^2^) are too low to reach the upper limit of lifetime ‘worst case scenario’; however, applied concentrations may simulate the mild risk of occupational exposures (lasting for 4–12 years). Lower doses applied for sfLM samples (~ 3.7 µg/cm^2^) corresponding with realistic ambient conditions showed no significant changes in any biological responses investigated, suggesting that the concentrations used here might be too low to activate the biological response. It is worth noting that lifetime exposures most probably occur repeatedly at long-time period at low doses. In the present study, cells were exposed only once to particles, thus more so reflecting an acute exposure scenario. However, according to the authors’ knowledge, only limited amount of the studies using the collected brake wear particles have been performed in a laboratory environment; therefore, it was difficult to estimate the most suitable concentration range for the present study (Kazimirova et al. 2016; Kukutschová et al. 2009; Malachova et al. 2016).

### Biological effects

Lung cell culture models are widely used to investigate the cellular response and the mechanisms of the interaction of inhaled particles with the cells, and are considered as an effective alternative towards the primary screening of a large range of different particle sizes and types (Nichols et al. 2014; Rothen-Rutishauser et al. 2008a). The 3D model of the human alveolar epithelial tissue barrier applied in the present study combines two types of primary human immune cells with alveolar type II epithelial cells and offers the possibility to study the interaction of individual cell types with the tested particles as well as the interaction among cell types (Rothen-Rutishauser et al. 2008b). The co-culture model was transferred to the ALI 24 h prior to the exposure to allow the epithelial cells to produce and release surfactant-based proteins at the apical side of the cells (Blank et al. 2006). Although different types of ALI cell exposure systems are available on the market (Lenz and Karg 2009; Loret et al. 2016; Paur et al. 2011), nebulisation of hydrophobic material with such a broad range of particle sizes was not possible and thus based on previous experimental approaches (Endes et al. 2014), the pseudo-ALI approach was applied resulting in thin layer of media with particles covering the surface of the co-culture model. This approach does not remove the surfactant as the cell surface was not washed before particles were added.

Elements detected in brake wear particles like iron, copper, titanium, etc. may lead to oxidative stress and (pro-)inflammatory response (Riediker et al. 2004; Schaumann et al. 2004). Ghio and Cohen (2005) suggested that a possible pathway to induce (pro-)inflammatory or (pro-)fibrotic response can be through inhaled PM, which interferes in the lungs with iron homeostasis. Oxidative stress resulting from the disruption of iron homeostasis in the cells is associated with the initiation and coordination of the (pro-)inflammatory response. Induction of a (pro-)inflammatory response was also observed in a recent study with rats following exposure to magnetite (Tada et al. 2012). Iron-based particles were detected only quantitatively in all tested samples; however, it was not possible to correlate the (pro-)inflammatory response with the presence of iron in the samples. Furthermore, it has been shown that TiO_2_ particles can elevate the TNF-α and IL-1β release as well as cytotoxicity and the production of reactive oxygen species (Xiong et al. 2013; Yazdi et al. 2010). No significant increase in IL-1β or TNF-α production was observed; however, a significant increase in IL-8 release compared to negative control for NAO sample was detected which could be related to the presence of TiO_2_ (see Table 1). Furthermore, it was observed that a slight increase (*p* > 0.05) in cell death occurred after exposure to NAO compared to exposure to the nL. Impaired cytoskeleton morphology following exposure to 2 mg/mL NAO sample was also seen. These findings, together with previous studies mentioned above (Xiong et al. 2013; Yazdi et al. 2010), lead the authors to hypothesize, that the presence of TiO_2_ in the brake formulations are directly related to the observed increased toxicity of brake wear particles.

## Conclusion

The exposures of different brake wear particle samples and size fractions using concentrations corresponding to ‘ambient conditions’ (lifetime doses) and acute high dose exposure conditions were successfully achieved. Low doses of different size fractions as well as brake wear particles with low-metallic formulation applied for 24 h did not activate a biological response of the lung cell co-culture model used. Contrastingly, sample with non-asbestos formula initiated a heightened (pro-)inflammatory response, and reduced cell viability together with an altered cell morphology. This can be attributed to the presence of anatase in the sample.

In summary, we showed that brake pad formulation can significantly differ and the released particles can induce different adverse effects in in vitro lung models. Commercially available methods were applied to generate wear particles; however, these finding are not representative for all materials and all braking scenarios which may occur and further research upon defining representative procedure for generation, sampling, and quantification of brake wear particles is needed.

## Electronic supplementary material

Below is the link to the electronic supplementary material.

Supplementary material 1 (TIF 761 KB)

Supplementary material 2 (DOCX 575 KB)
