# Supplementary figures and images for: Biological response of an in vitro human 3D lung cell model exposed to brake wear debris varies based on brake pad formulation
Source: Arch Toxicol. 2018 May 10;92(7):2339–51. doi: 10.1007/s00204-018-2218-8 (PMC6015608; doi:10.1007/s00204-018-2218-8)

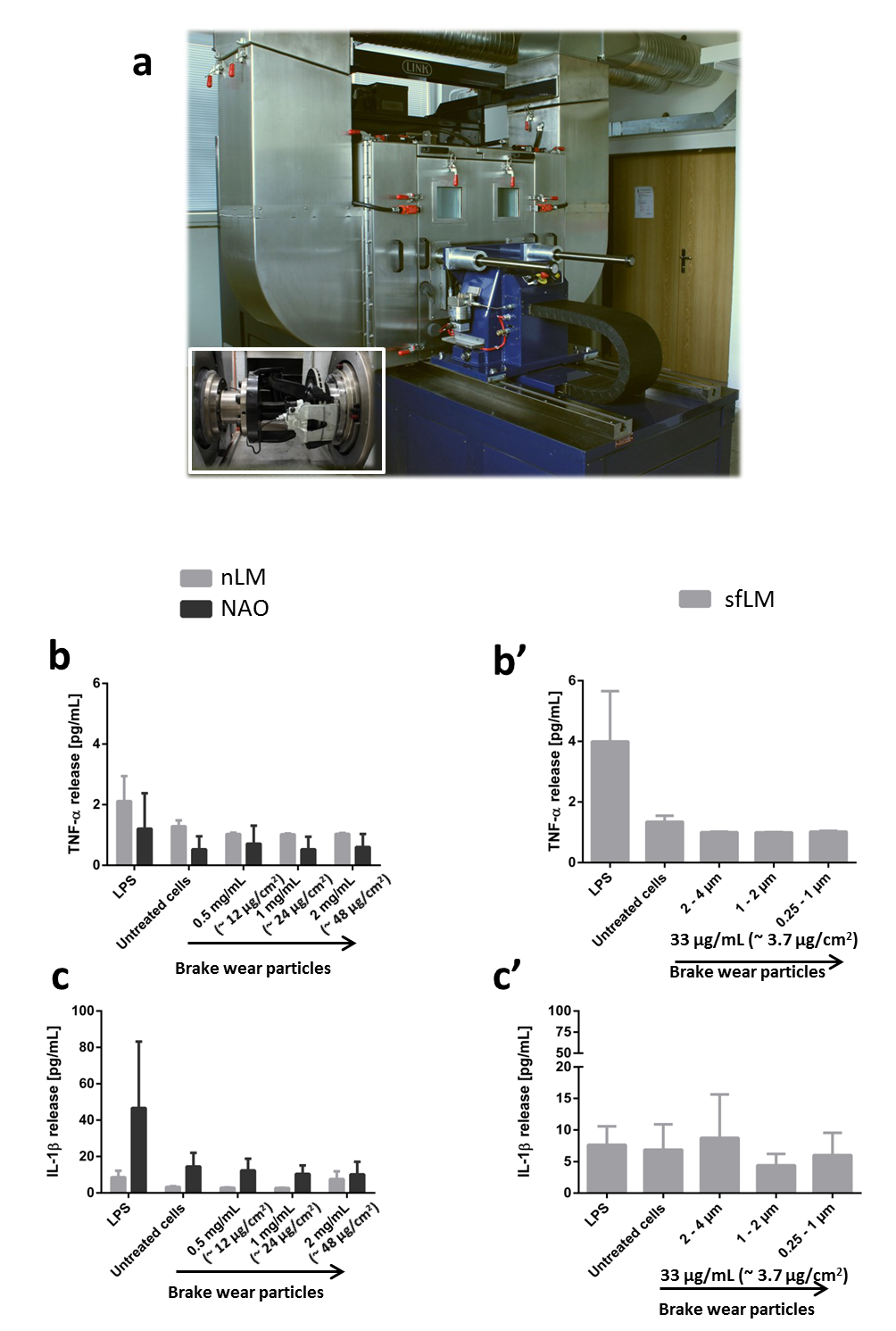

Supplement: Supplementary file 1 — Supplementary material 1 (TIF 761 KB) [file 204_2018_2218_MOESM1_ESM.tif]
