# Supplementary material for: Biological response of an in vitro human 3D lung cell model exposed to brake wear debris varies based on brake pad formulation
Source: Arch Toxicol. 2018 May 10;92(7):2339–51. doi: 10.1007/s00204-018-2218-8 (PMC6015608; doi:10.1007/s00204-018-2218-8)
Supplement: Supplementary file 2 — Supplementary material 2 (DOCX 575 KB) [file 204_2018_2218_MOESM2_ESM.docx]

**Supplementary material**

**Biological response of an *in vitro* human 3D lung cell model exposed to brake wear debris varies based on brake pad formulation**

Hana Barosova^1,2^, Savvina Chortarea^1,3^, Pavlina Peikertova^2^, Martin J.D. Clift^1,4^, Alke Petri-Fink^1,5^, Jana Kukutschova^2^, Barbara Rothen-Rutishauser^1*^

^1^ BioNanomaterials, Adolphe Merkle Institute, University of Fribourg, Fribourg, Switzerland

^2^ Nanotechnology Centre, VŠB-Technical University Ostrava, Ostrava, Czech Republic

^3^ Empa, Swiss Federal Laboratories for Materials, Science and Technology, Laboratory for Materials-Biology Interactions, St Gallen, Switzerland

^4^ *In Vitro* Toxicology Group, Swansea University Medical School, Swansea, Wales, UK

^5^ Chemistry Department, University of Fribourg, Fribourg, Switzerland

*Corresponding Author:

Prof. Dr. Barbara Rothen-Rutishauser: [barbara.rothen@unifr.ch](mailto:barbara.rothen@unifr.ch)

BioNanomaterials group

Adolphe Merkle Institute

Université de Fribourg, Chemin des Verdiers 4

CH-1700 Fribourg

**Co-culture model**

The co-culture model consisted of human epithelial type-II cells (A549 cell line), combined with primary human blood monocyte derived macrophages (MDMs) and dendritic cells (MDDCs) as described by Lehmann *et al.* (2010). A549 cells were cultured in Roswell Park Memorial Institute medium 1640 supplemented with 10 % fetal bovine serum, 1 % penicillin/streptomycin and 1 % L-Glutamine (sRPMI, all Gibco, USA) at 37° C, 5 % CO_2_. Epithelial cells were seeded at a density of 2.4x10^5^ cells/cm^2^, in BD Falcon cell culture inserts for 6-well or 12-well plates (high pore density transparent PET membrane, 3 μm diameter pore size and 4.2 cm^2^ growth area for 6-well inserts, 0.9 cm^2^ growth area for 12-well inserts respectively). Inserts were placed in culture plates (BD Biosciences, Switzerland) and cells were grown for 5 days under submerged conditions (*i.e.* medium in both upper (2 mL for 6-well insert, 1 mL for 12-well insert, respectively) and lower (3 mL for 6-well insert, 1,5 mL for 12-well insert, respectively) chamber of the insert). Peripheral human blood monocytes were isolated from human blood buffy coats (Blood Donation Service, Bern University hospital, Bern, Switzerland), as previously described by Lehmann *et al.* (2010) using CD14^+^ MicroBeads (Miltenyi Biotec GmbH, Germany) according to the manufacturer's protocol. Monocytes were cultured for 7 days prior assembling the co-culture at a density of 10^6^ cells/mL in supplemented RPMI 1640 medium. For the cell differentiation the growth factors (GM-CSF and IL-4 (both [10 ng/mL]) for MDDCs and M-CSF [10 ng/mL] for MDMs) were applied to the medium. The co-culture models were assembled, as previously described (Blank et al. 2007) resulting in cell density of 9.713x10^5 ±^1.497x10^5^ epithelial (A549) cells/cm^2^ on the apical part of the insert, 0.230x10^5^ ± 0.045x10^5^ MDMs/cm^2^ on the top of the co-culture model and 0.411x10^5^ ± 0.077x10^5^ MDDCs/cm^2^ on the basal part of the cell culture insert. After 24 h under submerged conditions the co-cultures were transferred to the ALI conditions, by removing the medium in the upper chamber and replacing the medium in the lower chamber with 1.2 mL of fresh culture medium in 6-well plate or 0.6 mL in 12-well plate respectively. The cells were then exposed to air for an additional 24 h prior to brake wear particle exposure being performed.

**Cell Viability**

*Propidium Iodide assay*

Exposed cells were incubated with Propidium Iodide (PI) (Annexin-V-FLUOS staining kit, Roche Diagnostics, Switzerland) to stain necrotic cells for 15 min at RT and then immediately analysed by flow cytometry (LSR Fortessa (3 laser, 4-blue-2-red-2-violet (405 nm (violet); 488 nm (blue); 640 nm (red)) BD Biosciences, Basel, Switzerland)). Untreated cells were incubated at – 80 °C for 1 h as a positive control to induce necrosis.

*Gating strategy for cell viability investigation*

Fluorescent signals were collected in logarithmic mode (4 decade logarithmic amplifier) and cell numbers per channel in linear mode. To identify each cell population, an electronic gate was placed around the side- and forward-scatter modes with 10’000 gated events acquired for each sample. The fluorescent amplifiers were adjusted to ensure that the negative cell population (i.e. non-fluorescent population) appeared in the first two logarithmic decades. An electronic marker was then placed at the limit of the negative control to express all positive cell populations in the final two logarithmic decades.

**Supplementary Figure 1**: Dynamometer Link M2800 used for brake wear particle generation with detail of the braking system (a). Quantification of TNF-α release for nLM and NAO samples (b) and for sfLM sample (b’). IL-1β release in cells exposed to nLM and NAO samples (c) and to sfLM sample (c’). Data are presented as mean ± standard error of the mean (SEM) (n=3). Data marked as (*) were considered statistically significantly increased compared to negative control (*p*<0.05). LPS: Lipopolysaccharide. **
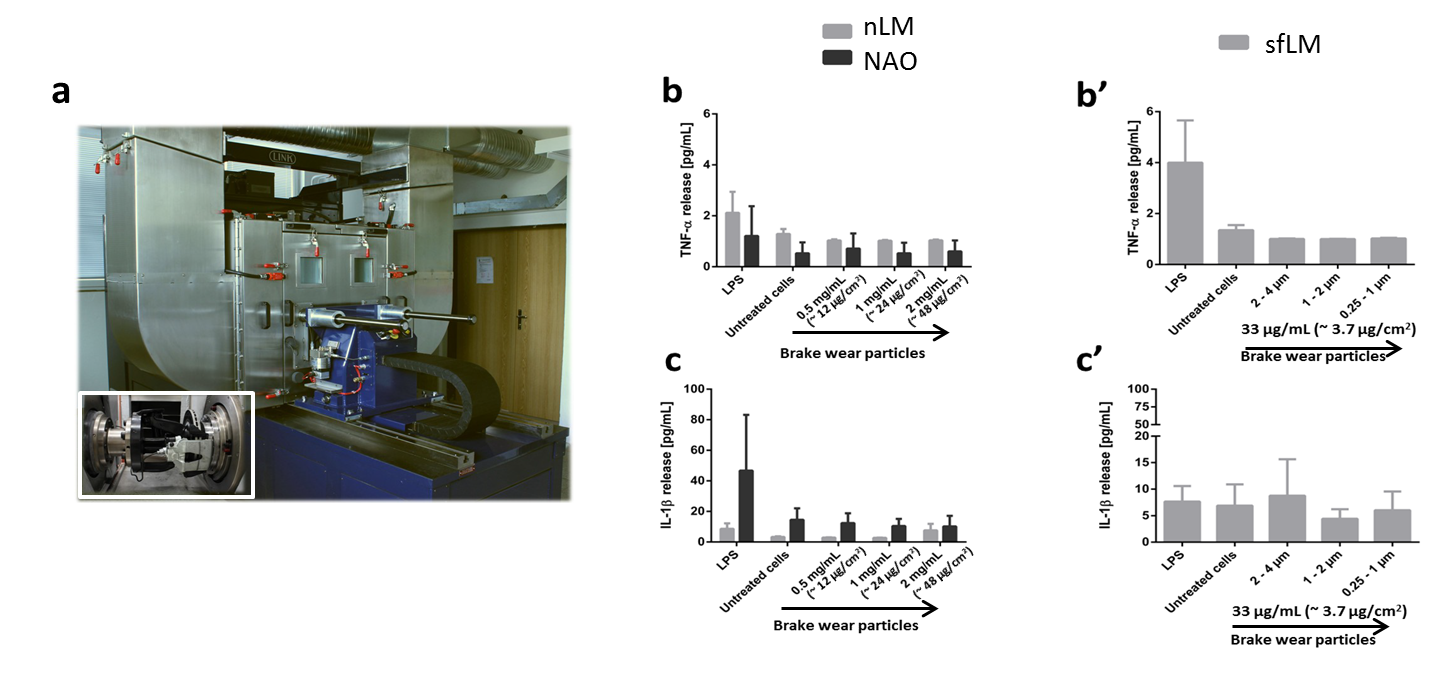
**

**References**

Blank F, Rothen-Rutishauser B, Gehr P (2007) Dendritic Cells and Macrophages Form a Transepithelial Network against Foreign Particulate Antigens. Am J Resp Cell Mol 36(6) doi:10.1165/rcmb.2006-0234OC

Lehmann A, Brandenberger C, Blank F, Gehr P, Rothen-Rutishauser B (2010) A 3D model of the human epithelial airway barrier. In: Yarmush ML LR (ed) Alternatives to animal testing. Artech House, p 239-260
